# *Cannabis* for Medical Use: Versatile Plant Rather Than a Single Drug

**DOI:** 10.3389/fphar.2022.894960

**Published:** 2022-04-25

**Authors:** Shiri Procaccia, Gil Moshe Lewitus, Carni Lipson Feder, Anna Shapira, Paula Berman, David Meiri

**Affiliations:** The Laboratory of Cancer Biology and Cannabinoid Research, Faculty of Biology, Technion- Israel Institute of Technology, Haifa, Israel

**Keywords:** cannabis, chemovar, phytocannabinoids, terpenoids, secondary metabolites

## Abstract

Medical *Cannabis* and its major cannabinoids (−)-*trans*-Δ^9^-tetrahydrocannabinol (THC) and cannabidiol (CBD) are gaining momentum for various medical purposes as their therapeutic qualities are becoming better established. However, studies regarding their efficacy are oftentimes inconclusive. This is chiefly because *Cannabis* is a versatile plant rather than a single drug and its effects do not depend only on the amount of THC and CBD. Hundreds of *Cannabis* cultivars and hybrids exist worldwide, each with a unique and distinct chemical profile. Most studies focus on THC and CBD, but these are just two of over 140 phytocannabinoids found in the plant in addition to a milieu of terpenoids, flavonoids and other compounds with potential therapeutic activities. Different plants contain a very different array of these metabolites in varying relative ratios, and it is the interplay between these molecules from the plant and the endocannabinoid system in the body that determines the ultimate therapeutic response and associated adverse effects. Here, we discuss how phytocannabinoid profiles differ between plants depending on the chemovar types, review the major factors that affect secondary metabolite accumulation in the plant including the genotype, growth conditions, processing, storage and the delivery route; and highlight how these factors make *Cannabis* treatment highly complex.

## Introduction

The past 2 decades have seen a major increase in the use of medical *Cannabis* as its therapeutic virtues are becoming better known and accepted (Bridgeman and Abazia, 2017). These therapeutic qualities were attributed to a naturally-occurring unique family of secondary metabolites termed phytocannabinoids. The most abundant and best-known phytocannabinoids are the psychoactive (−)-*trans*-Δ^9^-tetrahydrocannabinol (THC), which was first isolated and structurally elucidated by Mechoulam and colleagues in 1964 (Gaoni and Mechoulam, 1964); and cannabidiol (CBD), which was extracted in 1940 (Adams et al., 1940) and its full chemical structure was elucidated in 1963 by the same Mechoulam (Mechoulam and Shvo, 1963). CBD has been gaining interest since the 1980s when CBD oil was found to possess anti-epileptic properties (Consroe et al., 1982), and the CBD molecule was later shown to possess a wide range of therapeutic effects (Mechoulam et al., 2007; Zuardi, 2008). However, THC and CBD are just two of more than 140 distinctive phytocannabinoids that have been identified so far in different *Cannabis* plants (Hanuš et al., 2016; Berman et al., 2018).

The isolation of phytocannabinoids from the *Cannabis* plant has led to the discovery of endogenous cannabinoids (endocannabinoids, eCBs) in vertebrates (Devane et al., 1992). THC was found to bind a specific G-protein-coupled receptor, which was named cannabinoid receptor 1 (CB1) (Matsuda et al., 1990). A second receptor, which was named CB2, was identified by homology (Munro et al., 1993; Onaivi et al., 2006). Following the discovery of the receptors, their endogenous lipid ligands were identified. The first two and best-studied are N-arachidonoylethanolamine (anandamide) (Devane et al., 1992) and 2-arachidonoylglycerol (2-AG) (Mechoulam et al., 1995). These eCBs and their specific receptors, CB1 and CB2, form the classical endocannabinoid system (eCBS) (De Petrocellis and Di Marzo, 2009; Lu and Mackie, 2016), a ubiquitous neuromodulatory signaling system that has widespread functions in the brain and throughout the body. Since its inception, the term eCBS was expanded and now additional cannabinoid receptors, additional eCBs and cannabimimetic lipids as well as the enzymes involved in their synthesis and degradation are recognized as part of the extended eCBS (De Petrocellis et al., 2004; Mackie, 2008). Many of the pharmacological and therapeutic properties of phytocannabinoids rely on their interactions with the eCBS. The numerous and versatile effects of *Cannabis* result from the involvement of the eCBS in multiple processes. It regulates many physiological processes in health and disease (Di Marzo et al., 2004; de Fonseca et al., 2005). It is involved in the maintenance and homeostasis of many vital functions including immune response (Pandey et al., 2009), cardiovascular activity (Pacher and Steffens, 2009; Montecucco and Di Marzo, 2012), memory (Marsicano and Lafenêtre, 2009; Maroso et al., 2016; Lunardi et al., 2020) and pain sensation (Woodhams et al., 2015; Woodhams et al., 2017). This makes *Cannabis* treatment especially valuable since targeting the eCBS and its modulation by phytocannabinoids has been emerging as novel pharmacotherapy, with therapeutic potential suggested in a multitude of diseases affecting humans.

In the last decade, there has been a rapid growth in the discovery and use of pure THC, pure CBD and *Cannabis*-based extracts for various medical purposes. Results regarding the efficacy of *Cannabis*-based extracts are oftentimes inconclusive and sometimes even conflicting. That is because the effects of *Cannabis* extracts do not depend merely on the amount of THC and CBD (Maccarrone, 2020). *Cannabis* is a versatile plant rather than a single drug and importantly, studies involving pure THC or CBD do not reflect the potential benefits of full-spectrum extracts (Maayah et al., 2020b). For example, THC and CBD were both effective in reducing neuropathic pain in various mice and rat models (Comelli et al., 2008; Casey et al., 2017; King et al., 2017; Belardo et al., 2019; Abraham et al., 2020). However, the pain-relieving effects were enhanced by their combination (Casey et al., 2017; King et al., 2017). Moreover, a controlled high-CBD extract with additional secondary metabolites from the plant was more effective than purified CBD or THC at the same dose as in the extract (Comelli et al., 2008). In studies involving patients with multiple sclerosis, full-spectrum extracts demonstrated more beneficial effects for pain relief and reducing inflammation than pure THC and CBD (Maayah et al., 2020a; Maayah et al., 2020b). We have recently shown that both high-THC and high-CBD extracts were effective in reducing chronic pain, however, specific phytocannabinoid compositions were associated with more adverse effects (Aviram et al., 2021a). We also found *Cannabis* extracts effective in reducing migraine frequency, and here again, the presence of a few minor phytocannabinoids in the extracts made some more effective than others regardless of their THC or CBD content (Aviram et al., 2020b).

## Bioactive Secondary Metabolites From *Cannabis* as Therapeutic Agents

**FIGURE 1:**
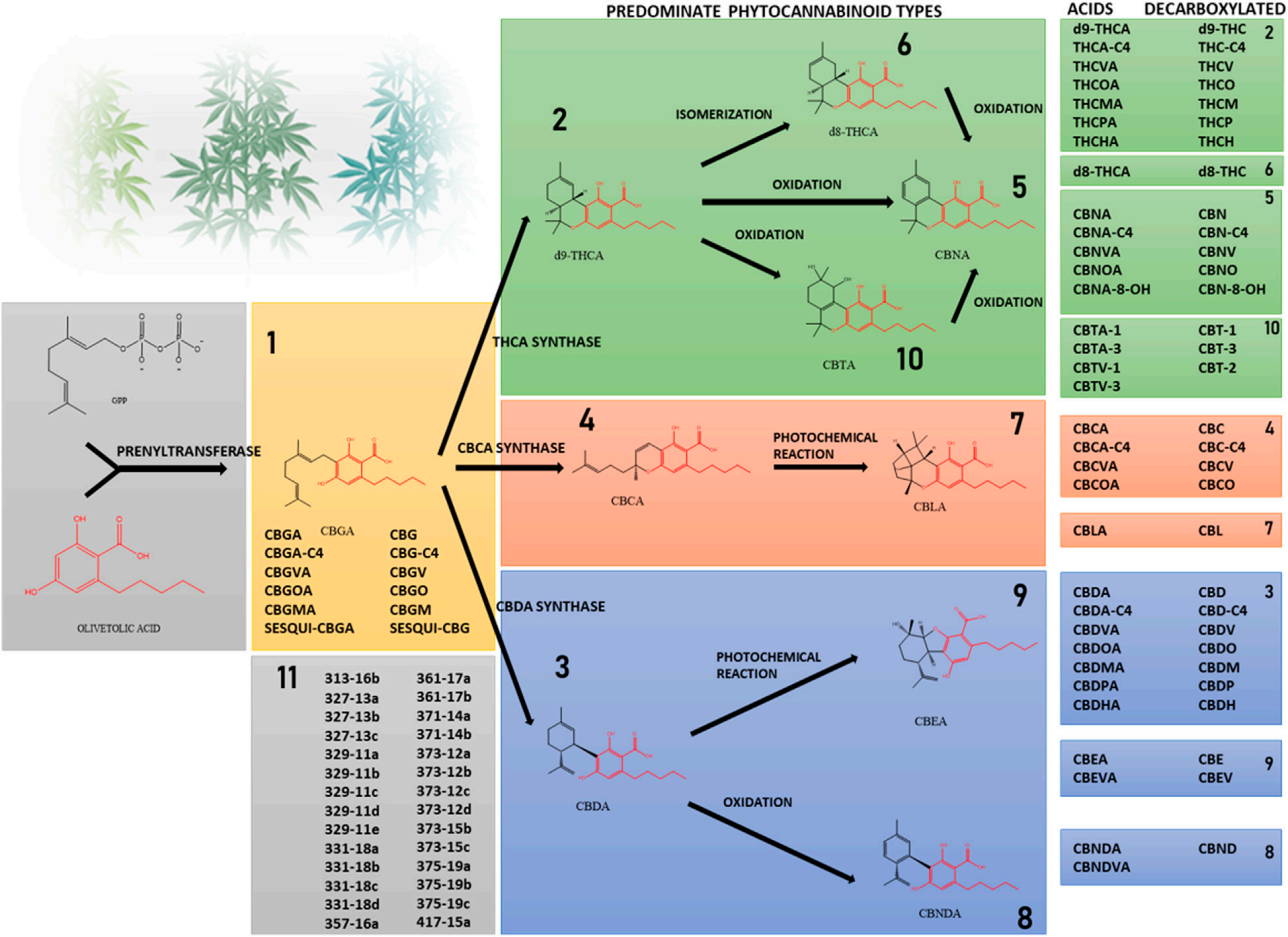
Phytocannabinoids are divided into subclasses according to their structure. Prenylation of olivetolic acid with the isoprenoid geranyl pyrophosphate forms CBGA. Less frequently, instead of olivetolic acid other molecules with different length alkyl side-chain serve as precursors. The acid forms THCA, CBDA and CBCA are synthesized in the *Cannabis* plant from CBGA. The neutral forms and other subclasses of phytocannabinoids are the result of chemical processes such as decarboxylation, isomerization and others. The shared core of olivetolic acid by the different subclasses is depicted in red. Subclass 11 includes phytocannabinoids identified by mass spectrometry in different *Cannabis* chemovars, whose structures have not been elucidated yet.

Phytocannabinoids are conventionally classified into 10 subclasses based on their chemical structure and an 11th miscellaneous types group (Figure 1) (Hanuš et al., 2016; Berman et al., 2018). They are lipophilic compounds biosynthesized by the convergence of two main plant pathways: the polyketide and the plastidial non-mevalonate-dependent isoprenoid (MEP) pathways. Phytocannabinoids are made of a resorcinyl core with a carboxyl group (COOH) on the aromatic ring, an alkyl side-chain of varying length that typically contains an odd number of carbon atoms (one to seven carbons), and a terpene moiety (Hanuš et al., 2016; Gülck and Møller, 2020). The most abundant type of phytocannabinoids in *Cannabis* are those with a pentyl side-chain (five carbons), with cannabigerolic acid (CBGA) as the first cannabinoid compound, made by the prenylation of olivetolic acid with the isoprenoid geranyl pyrophosphate (GPP) (Gülck and Møller, 2020). Other phytocannabinoid subclasses, including (−)-*trans*-Δ^9^-tetrahydrocannabinolic acid (THCA), cannabidiolic acid (CBDA) and cannabichromenic acid (CBCA) are derived from CBGA-type phytocannabinoids via specific enzymatic reactions (Berman et al., 2018). Thus, only these four subclasses are biosynthesized in the plant while the remaining subclasses are the result of different degradation routes and chemical processes such as oxidation, photochemical reaction, double bond isomerization, and others. The well-known neutral phytocannabinoids result from the decarboxylation of the acid compounds, where the carboxyl group is removed and carbon dioxide is released. In the less common cases, instead of olivetolic acid other molecules with different length alkyl side-chain serve as precursors. These undergo the same enzymatic and chemical reactions, resulting in a range of additional phytocannabinoids (Gülck and Møller, 2020) such as the three-carbon cannabigerovarinic acid (CBGVA), (−)-*trans*-Δ^9^-tetrahydrocannabivarinic acid (THCVA) and cannabidivarinic acid (CBDVA), or the seven-carbon (-)-trans-Δ^9^-tetrahydrocannabiphorol (THCP) and cannabidiphorol (CBDP) (Citti et al., 2019b) and others. Cannabinoid derivatives that were previously detected by MS methods are presented in Figure 1 (Berman et al., 2018; Citti et al., 2019a; Citti et al., 2019b; Linciano et al., 2020). Though initially considered unique to the *Cannabis* plant, other plant-derived natural products that are able to interact with ECS receptors were later discovered in other types of plants, such as *Radula marginata* and *Piper nigrum* (Gertsch et al., 2010; Russo, 2016).

In addition to phytocannabinoids, the other major active secondary metabolites of *Cannabis* are terpenes and terpenoids (generally termed terpenoids). Terpenes are naturally occurring volatile unsaturated hydrocarbon biomolecules built up by branched 5-carbon isoprene units, sharing the same isoprenoid precursor as phytocannabinoids. Terpenoids are modified terpenes that contain additional functional groups, usually varying oxygen arrangements or oxidation states. Monoterpenoids are built by two isoprene units (10 carbons) and sesquiterpenoids are built up by three isoprene units (15 carbons) (Shapira et al., 2019). Monoterpenoids and phytocannabinoids share the common biosynthetic precursor GPP and are both biosynthesized in the plastid, while sesquiterpenoids are synthesized in the cytosol from farnesyl pyrophosphate (Booth et al., 2020; Lipson Feder et al., 2021). Terpenoids are responsible for the fragrance and taste of plants as they are characterized by a strong and pleasant aroma (Gershenzon and Dudareva, 2007). Terpenoids are also suggested to have roles in protection from predation and attraction of pollinators. Terpenoids were shown to exert synergistic effects when combined with the phytocannabinoids in *Cannabis* and contribute crucially to its therapeutic effects (Downer, 2020; Ferber et al., 2020; Hanuš and Hod, 2020), and were also suggested to possess therapeutic effects of their own (Russo, 2011). Terpenoids are widely distributed in plants and a few are also present in other species including some animals and microorganisms (Gershenzon and Dudareva, 2007).

Various flavonoids are also found in *Cannabis* and may give the plant some of its exclusive medicinal benefits (Russo et al., 2003). Flavonoids are hydroxylated polyphenolic compounds consisting of two benzene rings linked via a heterocyclic pyran ring (Bautista et al., 2021). Three specific prenylated flavonoids, termed cannflavins A-C, are unique to *Cannabis* and show potent anti-inflammatory capabilities (Calzolari et al., 2017; Erridge et al., 2020). *Cannabis* plants produce additional kinds of secondary metabolites including various alkaloids, stilbenoids and others (Flores-Sanchez and Verpoorte, 2008a), but little is known regarding their biosynthesis and regulation and whether they possess any therapeutic value remains to be elucidated.

## New Analytical Approaches for Secondary Metabolites Profiling

It is the phytocannabinoids, terpenoids, flavonoids and other constituents in *Cannabis*, as well as their interplay, that determines the medicinal outcomes and adverse effects. As there is wide variability in their contents in different *Cannabis* plants (Delgado-Povedano et al., 2019; Bautista et al., 2021), there is a great need for their accurate chemical analyses that will help better understand the complexity and diversity of *Cannabis* compounds. Identification and quantification of phytocannabinoids and flavonoids can be achieved via gas chromatography (GC), either coupled to a flame ionization detector or a mass-spectrometer (MS). However, there are a few limitations to this method, as some analytes may not be sufficiently separated and decomposition is required for accurate quantification. Therefore, an alternative method using ultra-high-performance liquid chromatography with an ultraviolet detector (UHPLC/UV) and electrospray ionization-liquid chromatography/mass spectrometry (ESI-LC/MS) (Berman et al., 2018) allows for a high-resolution separation of components, without decomposition or derivatization prior to analysis. While UV detection is more appropriate for abundant components having analytical standards (such as THC, CBD and their corresponding acids), the use of mass spectrometry allows comprehensive identification and quantification of additional molecules, both abundant and rare. Additionally, MS and MS/MS analyses enable the identification of unknown molecules and their semi-quantification. Reference MS/MS data for identification of phytocannabinoids is available for labs and experts for putative identification (Berman et al., 2018). Terpenoids can be detected using static headspace gas chromatography-tandem MS (SHS-GC/MS/MS) (Shapira et al., 2019). Similar to phytocannabinoids, terpenoids with no commercially available analytical standards can still be semi-quantified relying on the calibration curves of molecules with standards and relying on both similar MS spectral characteristics and similar retention times (Lipson Feder et al., 2021).

## Strains, Cultivars and Chemovars

*Cannabis* is one genus with one species, *sativa* L. (ElSohly and Slade, 2005), which is sometimes divided into subspecies including in addition to *sativa* also *indica* and *ruderalis*. These *Cannabis* subspecies are divided into hundreds of different *Cannabis* cultivars and hybrids. Cultivar stands for cultivated variety, a plant that has been selected for cultivation. A *Cannabis* strain refers to plants reproduced asexually from a cultivar through clonal propagation. *Cannabis* cultivars worldwide vary significantly in their chemical compositions. Therefore, a *Cannabis* chemovar refers to the chemical profile of the plant and is considered a more useful classification in medicine (Hazekamp and Fischedick, 2012). Medical *Cannabis* has been divided into three phenotypic chemovar groups according to its content of THC and CBD: Type I which is THC-predominant, Type II in which the two are balanced and Type III which is CBD-predominant (Hazekamp and Fischedick, 2012).

**FIGURE 2:**
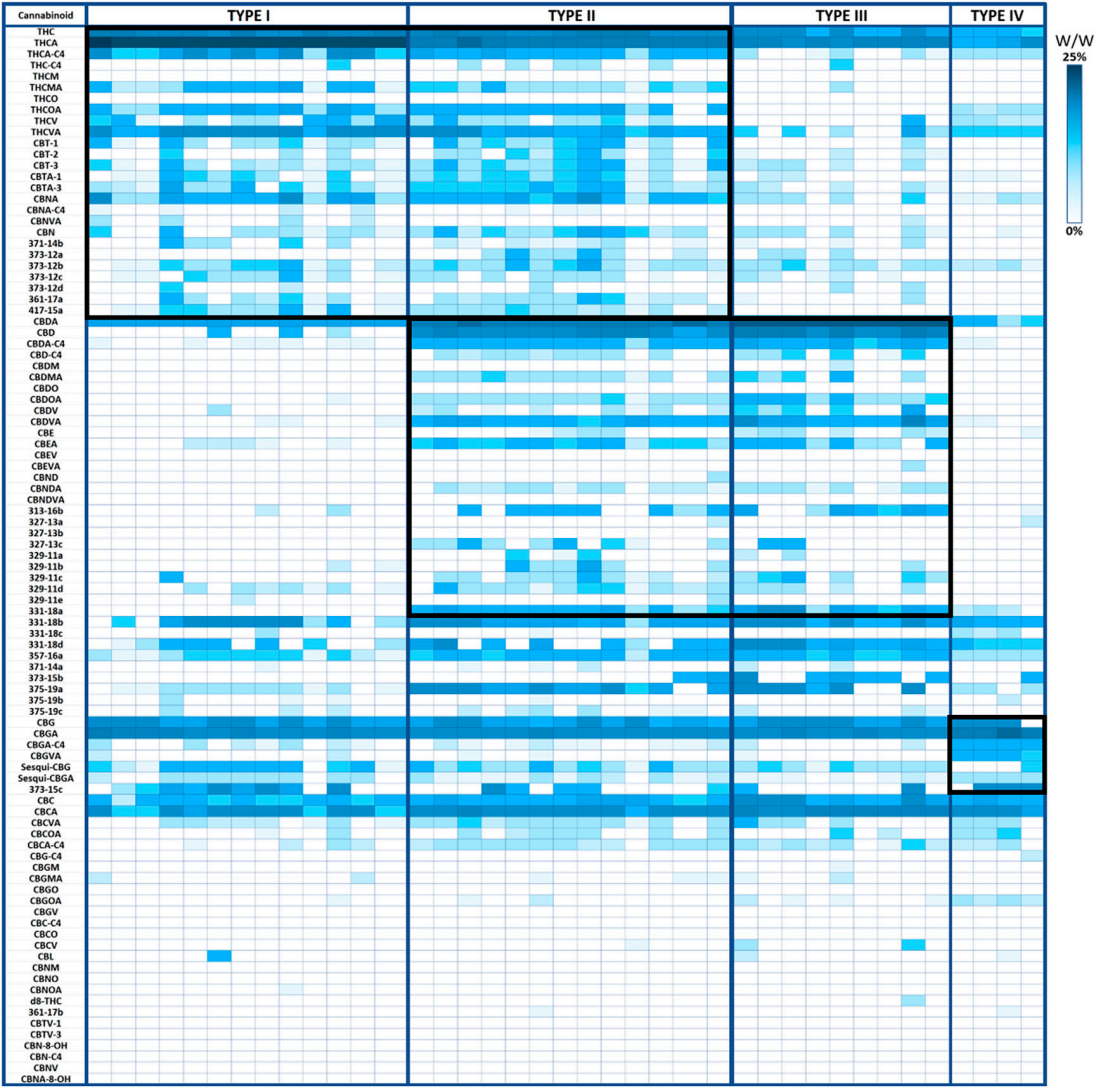
Minor phytocannabinoids are associated with Type I, Type III and Type IV chemovars. Heatmap presenting the concentration of phytocannabinoids (% weight per weight) divided by chemovars. Type I chemovars defined THCA >20% (*n* = 13), Type III chemovars defined CBDA >15% (*n* = 9), Type II defined THCA >4% and CBDA >10% (*n* = 13), type IV defined CBGA >6% (*n* = 4). Groups of unique phytocannabinoids are depicted by a surrounding black square.

**FIGURE 3:**
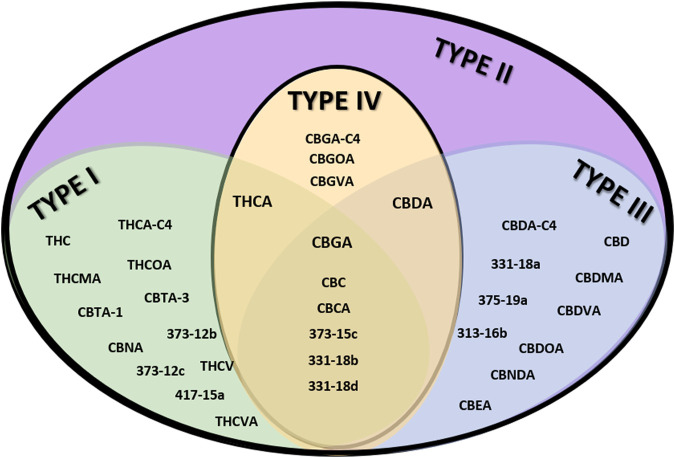
Venn diagram of the distribution of particular phytocannabinoids to specific chemovars. Examples of unique phytocannabinoids per chemovar type are shown in the appropriate subgroup.

From the genotypic perspective, *Cannabis* chemovar classification involves two codominant alleles on locus B, allele B_T_ is specific to THCA and allele B_D_ is specific to CBDA (De Meijer et al., 2003). Thus, Type I chemovar is B_T_/B_T_, Type III is B_D_/B_D_ and Type II is B_T_/B_D_. The nonfunctional allele B_0_ does not allow for the conversion of the precursor CBGA into THCA or CBDA, and is sometimes referred to as Type IV chemovar, which is CBGA-predominant. An independent gene at locus C codes for CBCA synthase that produces CBCA from CBGA (Hand et al., 2016). Studies showed that type I chemovar dominates the markets, but often it is not as beneficial as the other chemovars in achieving the desired symptom relief (Lewis et al., 2018; Aviram et al., 2020a; Aviram et al., 2021b). Moreover, the minor phytocannabinoid are not randomly distributed between the different chemovar types. As is shown in the heatmap presented in Figure 2, phytocannabinoids from cannabitriol (CBT) and cannabinol (CBN) families are more abundant in Type I chemovars, as they are predominantly the degradation products of THC. They can also be found in type II chemovars, though their concentration would generally be lower due to limitation in the amount of available precursor. Similarly, phytocannabinoids from cannabielsoin (CBE) family are more abundant in Type III chemovars as they are the degradation products of CBD and can also be found in type II chemovars to a lesser extent. Type-IV chemovars contain unique phytocannabinoids from the cannabigerol (CBG) family and high levels of phytocannabinoids from the cannabichromene (CBC) family, as CBCA synthase is intact. These selective distributions among chemovars are the result of metabolic pathways unique to either THC or CBD, which are not found in type IV chemovars. The distribution of particular phytocannabinoids according to chemovar is presented in Figure 3. Variations in the minor phytocannabinoid contents of different *Cannabis* extracts lead to varied effects on the eCBS, stressing the importance of their characterization in assessing cannabis effectivity (Berman et al., 2020). The high variability in the concentration of phytocannabinoid from 10 subclasses in their acidic and neutral forms in the inflorescences of 320 different cultivars is presented in Table 1.

**TABLE 1:** Variability of phytocannabinoids in 320 different cultivars.

Presented as concentration values (%w/w)		Max	Min	Average	Std dev
**1. Cannabigerol (CBG) type**					
Acids	CBGA	6.182	0.012	0.400	0.464
	CBGA-C4	0.028	0.000	0.001	0.002
	CBGVA	0.024	0.000	0.000	0.002
	CBGOA	0.004	0.000	0.000	0.000
	CBGMA	0.003	0.000	0.000	0.000
	Sesqui-CBGA	0.006	0.000	0.001	0.001
Neutrals	CBG	0.735	0.000	0.084	0.067
	CBG-C4	0.001	0.000	0.000	0.000
	CBGV	0.003	0.000	0.000	0.000
	CBGO	0.000	0.000	0.000	0.000
	CBGM	0.000	0.000	0.000	0.000
	Sesqui-CBG	0.042	0.000	0.008	0.006
**2. Δ** ^**9**^ **-trans-tetrahydrocannabinol (Δ** ^**9**^ **-THC) type**					
Acids	THCA	24.325	0.124	10.390	6.425
	THCA-C4	0.192	0.000	0.044	0.036
	THCVA	1.120	0.000	0.124	0.135
	THCOA	0.113	0.000	0.021	0.021
	THCMA	0.062	0.000	0.011	0.011
Neutrals	THC′	7.058	0.000	0.948	1.076
	THC-C4	0.062	0.000	0.003	0.008
	THCV	0.147	0.000	0.007	0.016
	THCO	0.000	0.000	0.000	0.000
	THCM	0.000	0.000	0.000	0.000
**3. Cannabidiol (CBD) type**					
Acids	CBDA	18.351	0.000	3.085	4.968
	CBDA-C4	0.094	0.000	0.009	0.016
	CBDVA	1.096	0.000	0.041	0.129
	CBDOA	0.053	0.000	0.003	0.007
	CBDMA	0.011	0.000	0.001	0.002
Neutrals	CBD	2.676	0.000	0.166	0.363
	CBD-C4	0.068	0.000	0.001	0.004
	CBDV	0.105	0.000	0.002	0.010
	CBDO	0.003	0.000	0.000	0.000
	CBDM	0.007	0.000	0.000	0.000
**4. Cannabichromene (CBC) type**					
Acids	CBCA	2.835	0.003	0.251	0.284
	CBCA-C4	0.006	0.000	0.001	0.001
	CBCVA	0.083	0.000	0.003	0.010
	CBCOA	0.018	0.000	0.001	0.002
Neutrals	CBC	0.830	0.000	0.034	0.055
	CBC-C4	0.001	0.000	0.000	0.000
	CBCV	0.014	0.000	0.000	0.001
	CBCO	0.000	0.000	0.000	0.000
**5. Cannabinol (CBN) type**					
Acids	CBNA	0.499	0.000	0.066	0.081
	CBNA-C4	0.002	0.000	0.000	0.000
	CBNVA	0.006	0.000	0.000	0.001
	CBNOA	0.001	0.000	0.000	0.000
	CBNA-8-OH	0.000	0.000	0.000	0.000
Neutrals	CBN	0.721	0.000	0.017	0.049
	CBN-C4	0.001	0.000	0.000	0.000
	CBNV	0.002	0.000	0.000	0.000
	CBNO	0.000	0.000	0.000	0.000
	CBNM	0.000	0.000	0.000	0.000
	CBN-8-OH	0.001	0.000	0.000	0.000
**6. Δ** ^**8**^ **-trans-tetrahydrocannabinol (Δ** ^**8**^ **-THC) type**					
Neutral	d8-THC	0.137	0.000	0.001	0.012
**7. Cannabicyclol (CBL) type**					
Neutral	CBL	0.040	0.000	0.000	0.003
**8. Cannabinodiol (CBND) type**					
Acids	CBNDA	0.014	0.000	0.001	0.002
	CBNDVA	0.000	0.000	0.000	0.000
Neutral	CBND	0.127	0.000	0.002	0.011
**9. Cannabielsoin (CBE) type**					
Acids	CBEA	0.056	0.000	0.004	0.008
	CBEVA	0.001	0.000	0.000	0.000
Neutrals	CBE	0.007	0.000	0.000	0.001
	CBEV	0.008	0.000	0.000	0.000
**10. Cannabitriol (CBT) type**					
Acids	CBTA-1	0.203	0.000	0.005	0.013
	CBTA-3	0.084	0.000	0.009	0.012
Neutrals	CBT-1	0.220	0.000	0.013	0.020
	CBTV-1	0.011	0.000	0.000	0.001
	CBT-3	0.172	0.000	0.009	0.015
	CBTV-3	0.010	0.000	0.000	0.001
	CBT-2	0.046	0.000	0.004	0.007

*n = 320* inflorescences from cultivars*;* results are concentration values of phytocannabinoids per plant (%w/w).

In addition, the *Cannabis* plant contains an overwhelming milieu of terpenoids, but only a limited number are currently reported and used for metabolic analyses of *Cannabis* chemovars (Shapira et al., 2019). Terpenoids content in different cultivars of *Cannabis* is highly variable, with some terpenoids being more associated with specific cultivars (Hillig, 2004; Casano et al., 2011). Studies that assessed terpenoid metabolism found the monoterpenoids limonene, β-myrcene, terpinolene and α-pinene, and the sesquiterpenoids β-caryophyllene and humulene, were abundant in the majority of *Cannabis* chemovars (Henry et al., 2018; Lewis et al., 2018). Some terpenoids were predominantly found only in Type I chemovars and others only in Type III, suggesting joint metabolic pathways and chemovar-specific aroma and effects (Lewis et al., 2018). Table 2 summarizes the variability of monoterpenoids and sesquiterpenoids in 79 distinct *Cannabis* inflorescences (out of the 320 described for phytocannabinoids in Table 1).

**TABLE 2:** Variability of terpenoids in 79 different cultivars.

Compound	Max (ppm)	Min (ppm)	Average (ppm)	Std dev (ppm)	V (%)
α-Pinene	1903.4	2.3	181.7	357.5	196.8
Camphene	161.6	1.7	18.3	34.0	186.3
Sabinene	3.7	0.0	0.9	1.1	126.3
β-Pinene	1705.3	2.2	132.1	259.9	196.8
β-Myrcene	>2,706	5.1	444.3	706.4	159.0
3δ-Carene	530.3	0.0	10.1	62.7	622.3
α-Phellandrene	701.5	0.0	14.0	80.5	574.7
α-Terpinene	379.0	0.0	14.9	51.1	343.1
Limonene	>2,760	2.7	247.5	577.7	233.5
β-Phellandrene	421.1	0.0	16.9	55.8	330.6
*cis*-Ocimene	101.6	0.0	4.3	12.9	302.4
Eucalyptol	63.6	0.0	7.3	11.9	162.9
p-Cymene	28.7	0.0	2.2	4.3	192.5
*trans*-Ocimene	1,648.5	0.0	62.9	237.6	377.8
γ-Terpinene	512.2	1.5	16.4	60.7	369.2
Terpinolene	>2,433	2.4	96.2	394.4	410.1
Linalool	1,204.4	0.0	214.1	249.8	116.7
Fenchone	68.0	0.0	8.9	12.5	139.5
Fenchol	953.7	0.0	118.1	145.4	123.1
C_10_H_18_O-154 (99/93/79/121)-1*	222.6	0.0	25.8	43.3	168.3
C_10_H_18_O-154 (99/93/79/121)-2*	29.3	0.0	0.4	3.3	0.0
Menthol	62.1	0.0	5.6	12.9	230.7
Borneol	941.0	0.0	59.8	123.7	206.7
Camphor	20.4	0.0	1.0	2.6	257.5
Terpinen-4-ol	149.3	0.0	17.9	29.3	163.2
α-Terpineol	1,027.8	0.0	98.1	148.3	151.1
Citronellol	129.1	0.0	12.7	25.9	204.3
Nerol	26.2	0.0	2.5	5.4	218.6
Geraniol	93.8	0.0	4.1	14.1	347.1
Bornyl acetate	37.2	0.0	2.8	6.6	236.5
α-Cubebene*	8.2	0.0	2.2	1.9	110.8
Isoledene	7.7	0.0	0.1	0.9	885.1
Cyclosativene	11.6	0.0	0.2	1.3	728.5
Ylangene*	74.5	0.0	5.6	9.8	176.4
α-Copaene*	12.9	0.0	2.2	2.4	118.0
α-Funedrene	1.8	0.0	0.9	0.5	146.0
7-epi-Sesquithujene*	76.3	0.0	13.3	15.4	116.3
C_15_H_24_-204 (105/(120+119)/161)*	13.4	0.0	2.8	3.2	114.7
Sativene	2.5	0.0	1.0	1.1	106.4
β-Cubebene*	5.2	0.0	0.1	0.6	886.1
Sesquithujene*	116.0	0.0	14.9	17.6	118.4
β-Isocomene*	41.3	0.0	6.7	8.5	126.1
α-Santalene*	71.5	0.0	7.7	11.0	142.8
*cis*-α-Bergamotene*	23.6	0.0	3.9	4.4	113.2
α-Cedrene	3.9	0.0	1.1	1.1	104.7
*trans*-α-Bergamotene*	63.6	0.0	7.0	13.8	196.5
β-Caryophyllene	>3,631.5	9.0	670.8	781.0	116.4
Geranyl acetate	1.8	0.0	0.4	0.6	144.7
β-Cedrene	18.5	0.0	1.0	2.9	284.5
α-Guaiene*	567.3	0.0	58.6	107.6	183.7
γ-Elemene*	161.1	0.0	11.9	24.1	202.5
Aromadendrene	8.3	0.0	1.9	2.2	120.3
β-Santalene*	39.2	0.0	3.0	5.7	187.8
Guaia-6,9-diene*	65.4	0.0	7.3	12.5	171.3
*trans*-β-Farnesene	617.3	3.2	44.5	71.7	161.4
C_15_H_24_-204 (69/91/105/161)*	25.7	0.0	3.9	6.3	160.6
C_15_H_24_-204 (91/105/161)*	302.3	0.0	12.3	34.2	279.0
C_15_H_24_-204 (161/105/133/91)*	44.4	0.0	9.1	11.0	121.5
C_15_H_24_-204 (105/91/133/161/189)*	44.2	0.0	9.5	11.0	115.9
α-Humulene	2,134.2	12.5	255.8	283.9	111.0
Alloaromadendrene	104.1	0.0	12.0	17.3	143.7
Acoradiene*	10.5	0.0	1.2	2.3	195.4
C_15_H_24_-204 (105)-1*	35.4	0.0	8.3	10.3	123.6
γ-Curcumene*	432.5	0.0	9.7	48.6	500.5
C_15_H_24_-204 (189/133)-1*	101.4	0.0	19.9	25.9	130.2
Sesquisabinene*	56.8	0.0	5.0	8.8	173.6
γ-Muurelene*	60.9	0.0	7.6	10.7	140.2
α-Amorphene*	27.0	0.0	6.7	7.5	112.5
Aristolochene*	14.6	0.0	1.8	2.7	152.4
Germacrene D*	28.3	0.0	4.3	7.7	180.4
β-Chamigrene	16.3	0.0	0.5	2.6	488.0
C_15_H_24_-204 (189/133)-2*	196.3	0.0	44.0	46.9	106.6
C_15_H_24_-204 (119/93/161)*	28.1	0.0	3.6	5.7	157.4
α-Selinene*	92.0	0.0	16.7	21.1	126.4
Ledene	6.0	0.0	0.1	0.7	688.9
α-Curcumene	69.9	0.0	9.8	17.6	180.2
Valencene	402.8	0.0	26.6	80.5	302.9
β-Selinene*	716.9	0.0	133.0	184.3	138.5
α-Farnesene*	88.7	0.0	6.8	13.4	196.4
β-Bisabolene*	663.2	0.0	42.7	87.0	204.0
δ-Guaiene*	560.0	0.0	47.2	96.5	204.3
C_15_H_24_-204 (119/161/105/134)*	32.6	0.0	6.2	7.8	125.4
β-Curcumene	27.9	0.0	5.0	6.6	130.0
Dihydroagarofuran*	15.3	0.0	2.2	3.2	145.7
C_15_H_24_-204 (similar Germarcene B)*	32.7	0.0	8.5	9.5	111.7
Sesquicineole*	135.1	0.0	9.4	16.5	175.2
Eremophilene*	38.6	0.0	9.0	11.5	128.6
β-Sesquiphellandrene*	77.7	0.0	9.3	14.0	149.9
γ-Cadinene*	22.3	0.0	4.5	5.8	128.7
δ-Cadinene*	27.4	0.0	7.0	6.7	95.8
C_15_H_24_-204 (105)-2*	28.9	0.0	7.3	8.7	118.6
α-Panasinsene*	31.3	0.0	1.3	3.9	288.6
*trans*-α-Bisabolene*	512.1	0.0	86.2	97.9	113.6
Selina-3,7 (11)-diene*	>1,334.1	0.0	249.3	361.5	145.0
trans-Nerolidol	1,637.2	0.0	102.1	240.3	235.3
Germacrene B*	923.0	0.0	25.7	107.1	417.0
Globulol	31.2	0.0	0.5	3.6	698.6
Guaiol	>2099	0.0	568.1	765.5	134.7
Caryophyllene oxide	>1890	11.2	308.5	488.4	158.3
α-epi-7-epi-5-Eudesmol*	319.1	0.0	30.8	47.5	154.2
C_15_H_26_O-222 (similar γ-Eudesmol)*	>2099	0.0	541.8	751.1	138.6
Selina-6-en-4-ol*	180.3	0.0	29.8	46.0	154.2
γ-Eudesmol*	>1,588	0.0	296.1	478.4	161.6
Hinesol*	196.1	0.0	33.1	39.2	118.5
C_15_H_26_O-222 (105/161/59)-1*	496.1	0.0	63.8	108.8	170.5
Agarospirol*	158.1	0.0	14.7	26.2	178.5
C_15_H_26_O-222 (105/161/59)-2*	812.2	0.0	78.3	135.2	172.5
C_15_H_26_O-222 (59/81/107/149/161)*	566.6	0.0	66.1	96.2	145.6
α-Eudesmol*	>1,588	0.0	377.3	541.5	143.5
β-Eudesmol	>1,588	0.0	434.2	573.0	132.0
7-epi-α-Eudesmol*	573.7	0.0	61.0	108.5	177.9
Bulnesol*	>2099	0.0	159.9	318.0	198.9
α-Bisabolol	>3,791	0.0	1,515.7	1,592.2	105.0
**Total monoterpenoids [ppm]**	18,783.3	44.5	1842.0	2,896.2	157.2
**Total monoterpenoids [%]**	1.88	0.00	0.18	0.29	0.02
**Total sesquiterpenoids [ppm]**	25,135.2	147.6	6,678.2	5,089.5	76.2
**Total sesquiterpenoids [%]**	2.51	0.01	0.67	0.51	0.01
**Total terpenoids [ppm]**	26,501.4	196.1	8,520.2	6,047.6	71.0
**Total terpenoids [%]**	2.65	0.02	0.85	0.60	0.01

*n* = 79 inflorescences from cultivars; ppm–parts per million, > values above upper limit of detection, % represents concentration values of terpenoids per plant, * terpenoids that were semi-quantified.

Each *Cannabis* cultivar contains a different profile of more than 500 secondary metabolites (ElSohly and Slade, 2005; Andre et al., 2016; Berman et al., 2018; Piper, 2018). The fact that hundreds of different *Cannabis* cultivars and hybrids exist worldwide, varying significantly in their chemical compositions, makes *Cannabis* treatment highly complex. Moreover, sometimes the outcome of treatment with medical *Cannabis* depends on the way its secondary metabolites act together synergistically, in a mechanism first described by Ben-Shabat and Mechoulam for eCBs (Ben-Shabat et al., 1998) and later postulated by Russo as the ‘entourage effect’ for phytocannabinoids (Russo, 2011). Thus, phytocannabinoids that are found together in a *Cannabis* chemovar modulate each other’s activity and thus the overall effect. The entourage effect postulates that the presence of minor phytocannabinoids, terpenoids and other plant metabolites contributes to the overall response in a way that significantly modulates the effects of the main active components, THC and CBD, and thereby produces more potent or more selective effects. Several studies have shown whole extracts or a combination of THC and CBD, with either each other, minor phytocannabinoids or terpenoids, are more effective than the corresponding major phytocannabinoid in producing the same response (Russo, 2011; Velasco et al., 2016; Blasco-Benito et al., 2018; Baram et al., 2019; Namdar et al., 2019; Ferber et al., 2020). However, other studies did not find evidence that common terpenoids can bind eCBS receptors or modulate the effect of phytocannabinoids on the receptors (Santiago et al., 2019; Finlay et al., 2020; Heblinski et al., 2020). A better understanding of the different components in *Cannabis* and the way they act together is required to fully utilize its therapeutic potential to the fullest.

## Pre- and Post-Harvest Conditions

The concentrations of the different compounds in the plant depend on many factors. There is a strong genotypic influence on the composition of secondary metabolites in different *Cannabis* chemovars (Aizpurua-Olaizola et al., 2016; Welling et al., 2018; McGarvey et al., 2020). However, a very large variation exists also in the profiles of genetically identical plants grown under different conditions (De Backer et al., 2009). For example, we previously showed the differences in phytocannabinoids profiles of a high-CBD *Cannabis* chemovar that was used to treat refractory childhood epilepsy in Israel (Berman et al., 2018). While the genetically identical plants from four different greenhouses were planted and harvested in the same way and at the same time, and considered as the same treatment, their CBDA contents were similar but they portrayed substantial differences in many other phytocannabinoids.

In addition to the genetic variety, many environmental factors affect the composition of the secondary metabolites in the *Cannabis* plant (Tang et al., 2016). These include growth conditions such as humidity, light quality and intensity, CO_2_ concentration and mineral nutrition (Chandra et al., 2008; Chandra et al., 2017; Bernstein et al., 2019a). The tissue type is also an important factor as within the plant there is a location- and organ-specific distribution of the active secondary metabolites (Happyana et al., 2013; Bernstein et al., 2019a; Bernstein et al., 2019b). Phytocannabinoids are synthesized in glandular trichomes that are located in the highest density on the inflorescences of unfertilized female plants (Lipson Feder et al., 2021), and their accumulation varies in the different aerial parts (flowers, fan leaves, inflorescence leaves, stalk and stem). Accumulation patterns also depend on the age of that part (Flores-Sanchez and Verpoorte, 2008b; Hazekamp and Fischedick, 2012). A study that tested phytocannabinoid and terpenoid content in the plant from the rooting until the end of the flowering stage (Aizpurua-Olaizola et al., 2016) found that the accumulation of some major phytocannabinoids and monoterpenoids requires longer growth time in plants from Type II and Type III chemovars than in Type I. The functional roles of phytocannabinoids and terpenoids *in planta* are still not fully elucidated as well as the biosynthesis pathways involved in their production and the mechanisms of localization and secretion. Cannflavins accumulation also varies depending on the part of the plant, they are found in most parts, including the leaves and inflorescences, but are undetectable in roots and seeds (Flores-sanchez and Verpoorte, 2008b). Interestingly, all three cannflavins A-C were found in greater amounts in genetically identical *Cannabis* plants grown at a higher altitude (Giupponi et al., 2020).

Importantly, the composition and concentration of the different secondary metabolites are also affected by harvest time (Happyana and Kayser, 2016) and change over time postharvest as a result of different degradation routes, depending on the storage conditions and its duration (Trofin et al., 2011; Jin et al., 2019; Zamengo et al., 2019; Milay et al., 2020). The concentrations of terpenoids rapidly decline in storage due to their volatile nature (Milay et al., 2020). For phytocannabinoids, one of the main processes that occur during storage is decarboxylation. Over time due to heat and light, the acidic forms undergo spontaneous decarboxylation, but the extent of which is not uniform. For example, THC is the neutral counterpart of THCA. However, THCA is only partially converted to THC and to varying degrees (Dussy et al., 2005; Jung et al., 2009). THCA has different biological characteristics than THC, it is not psychoactive and has a distinctive pharmacological activity (Moreno-Sanz, 2016). Several studies reported on the therapeutic activities of phytocannabinoids in their acidic form. For example, CBDA was found to be a more potent antiemetic and anticonvulsant agent than CBD *in-vivo* (Bolognini et al., 2013; Anderson et al., 2019), as well as a better inhibitor of breast cancer cell migration *in-vitro* (Takeda et al., 2012). Therefore, the relative ratio between THCA and THC, or between CBDA and CBD, has a therapeutic implication that has yet to be fully elucidated. For phytocannabinoids, the content of CBN is used as a marker for *Cannabis* aging, however, it is not a relevant marker in Type III chemovars (Milay et al., 2020) as it is formed mainly via the oxidation of THC or the decarboxylation of its acidic form cannabinolic acid (CBNA), which in turn rises from the oxidation of THCA.

In a study that tested the optimal postharvest processing, solvents and a range of temperatures, it was concluded that the conditions that best preserved the composition of the secondary metabolites relative to their pre-storage composition were unextracted whole inflorescences at 4°C (Milay et al., 2020). The duration of storage, as well as of drying and curing before storage, varies greatly; as a consequence, a very large variation exists in the phytocannabinoid and terpenoid profiles of *Cannabis* chemovars that are considered the same.

## Delivery Routes

As the active biomolecules in *Cannabis* such as phytocannabinoids are highly lipophilic and therefore present poor oral bioavailability, various administration routes have been investigated for the therapeutic use of *Cannabis*, including the pulmonary, sublingual, oral, dermal and rectal routes (Bruni et al., 2018). Currently, the common administration routes of whole-plant and plant-derived *Cannabis* products are either by inhalation (smoking or vaporization) or ingestion of edibles (Hazekamp et al., 2013; Bridgeman and Abazia, 2017). However, the pharmacokinetics and the effects observed with *Cannabis* administration vary significantly as a function of the delivery route, formulation, and the ratios between the multiple active compounds. For example, the acidic pH of the stomach further reduces bioavailability via the oral route (Grotenhermen, 2003). Moreover, to be used via the oral or sublingual routes, the active secondary metabolites in the plant must be extracted. The extraction method and choice of extracting solvent affect the secondary metabolite profile (Křížek et al., 2018), a phenomenon which was shown for phytocannabinoids (Turner et al., 2017; Namdar et al., 2019), terpenoids (Shapira et al., 2019) and flavonoids (Isidore et al., 2021).

Inhalation provides a rapid and efficient method of drug delivery. Symptom relief is immediate and effective, the dosage can be more controlled than via the alternative routes, and a lower dose can be used to get the desired effect (Foster et al., 2019). However, inhalation has several considerable disadvantages; it leads to high and prompt peak plasma concentration of cannabinoids such as THC and CBD post inhalation (Huestis, 2005), causing a more intense and shorter-lasting effect than other routes, which in turn may be associated with higher toxicity (Dinis-oliveira, 2016). Smoking is associated with health risks and the formation of toxic and carcinogenic substances during combustion (Gates et al., 2014), vaporizers do not heat *Cannabis* to the point of combustion (i.e., less than 170–190°C), but still induce heat and expose to a variety of undesirable chemicals (Grotenhermen, 2003; Shiplo et al., 2016). All the bioactive molecules of *Cannabis* are susceptible to degradation processes such as decarboxylation when *Cannabis* is heated above 120°C by smoking or vaping, as well as by cooking (Dussy et al., 2005).

The pharmacokinetics of the current consumption options modulates and limits the therapeutic bioavailability of *Cannabis* metabolites. For example, when THC is ingested rather than inhaled, it is metabolized by the liver before entering the bloodstream and hydroxylated to 11-hydroxy-THC, which is equally potent (Perez-Reyes et al., 1972; Hollister, 1974) or might be even more potent than THC (Christensen et al., 1971; Schwilke et al., 2009), and then further oxidized to the inactive metabolite 11-COOH-THC. This makes the consideration of the *Cannabis* delivery system vital for its effective administration and treatment (Uziel et al., 2020).

New analytical approaches now allow for more accurate profiling of *Cannabis* metabolites both in the plant itself and in the tissues they affect, allowing to better investigate their disposition over time by the body of the organism (Huestis, 2007). Many of the alternative routes to inhalation and digestion are aimed at improving the bioavailability via avoiding degradation with first-pass metabolism by the liver. Other delivery routes that have yet to be explored are intravenous, intramuscular and intranasal. Emulsions via nanotechnology advances are also aimed at improving the bioavailability of the active molecules in *Cannabis* (Holgado et al., 2017; Adusumilli et al., 2021).

## Discussion and Future Perspectives

The use of medical *Cannabis* is ever increasing in the treatment of numerous conditions as it has been proven to be both effective and safe, but the *Cannabis* plant contains more than 500 different components, each with potential therapeutic qualities. The components of *Cannabis* act together, hitting several targets at once and mutually enhancing each other’s activity so that the overall outcome is greater than that of their additive effect. The concentrations and combinations of the various secondary metabolites, including the way they complement each other, determine both the final medicinal response and adverse effects.

*Cannabis* can treat a multitude of very different conditions as it exerts its effects via the ECS, which is involved in many physiological processes. *Cannabis* treatment can be personalized to both the condition and the person to improve treatment outcomes while also reducing the drug load and minimizing the adverse effects. Most patients do not receive *Cannabis*-based medication but rather whole plants or extracts that contain many active bio-compounds in different proportions. Each has a different profile of components, undergoing different drug interactions. It is still unknown which molecules in the whole extract are responsible for its overall effect and via which ECS receptors, effectors and metabolic pathways. Further research is needed to find which whole extracts or specific molecules are best suited to treat a given condition.

Physicians and patients require more information to guide them in choosing the most appropriate cultivar or molecules, in the correct dose and via the optimal delivery route. The number of studies that tested different cannabinoids or tried to recognize the specific bioactive molecules from whole extracts is low and should be addressed to fulfill the full potential of *Cannabis* and improve human health.
